# Relative dose intensity of first-line chemotherapy and overall survival in patients with advanced non-small-cell lung cancer

**DOI:** 10.1007/s00520-019-04875-1

**Published:** 2019-06-07

**Authors:** Jeffrey Crawford, Neelima Denduluri, Debra Patt, Xiaolong Jiao, Phuong Khanh Morrow, Jacob Garcia, Richard Barron, Gary H. Lyman

**Affiliations:** 1 Duke University Medical Center, Trent Drive, Duke South, 25177 Morris Building, Durham, NC 27710 USA; 2 Virginia Cancer Specialists, US Oncology Network, Arlington, VA USA; 3 McKesson Specialty Health, The Woodlands, TX USA; 4 Amgen Inc., Thousand Oaks, CA USA; 5 Fred Hutchinson Cancer Research Center and the University of Washington, Seattle, WA USA

**Keywords:** Lung cancer, Chemotherapy, Retrospective studies, Community health services

## Abstract

**Purpose:**

The effects of chemotherapy dose intensity on survival in patients with advanced non-small-cell lung cancer (NSCLC) are poorly understood. We retrospectively analyzed dose delays/reduction, relative dose intensity (RDI), and the association between chemotherapy intensity and survival in advanced NSCLC.

**Methods:**

This retrospective cohort study included adults with advanced lung cancer who received first-line myelosuppressive platinum-based chemotherapy (January 2007–December 2010) in ~ 230 US Oncology Network community practices. Dose delays ≥ 7 days, dose reductions ≥ 15%, and RDI relative to standard regimens were described. Overall survival (OS) was measured using Kaplan-Meier and Cox proportional hazard (PH) models.

**Results:**

Among 3866 patients with advanced NSCLC, 32.4% experienced dose delays ≥ 7 days, 50.1% experienced dose reductions ≥ 15%, and 40.4% had RDI < 85%. Reduced RDI was also common regardless of baseline ECOG PS (ECOG PS ≥ 2, 56.2%; ECOG PS 0, 33.6%) and tumor subgroup (squamous cell carcinoma, 52.2%; adenocarcinoma, 36.0%). When stratified by chemotherapy intensity measures, significant OS differences were observed only for dose delays. Median (95% CI) OS was 1.02 years (0.96–1.12) for dose delays ≥ 7 days and 0.71 years (0.66–0.77) for dose delays < 7 days. In multivariable Cox PH analysis, dose delays ≥ 7 days (HR = 0.71; 95% CI = 0.63–0.80) and RDI ≥ 85% (HR = 1.18; 95% CI = 1.05–1.32) were significantly associated with decreased mortality.

**Conclusions:**

Dose delays, dose reductions, and reduced RDI were common, and dose delays ≥ 7 days and high RDI were significantly associated with decreased mortality. These results can help identify potential risk factors and characterize the effect of chemotherapy dose modification strategies on mortality.

**Electronic supplementary material:**

The online version of this article (10.1007/s00520-019-04875-1) contains supplementary material, which is available to authorized users.

## Introduction

Lung cancer is the second most common malignancy in the USA, with an annual incidence of 55.8 per 100,000 [1]. An analysis of the National Cancer Institute’s Surveillance Epidemiology and End Results (SEER) program reported that in 2010, most patients with non-small-cell lung cancer (NSCLC) were in an advanced stage at diagnosis: 8% of patients were in stage 3A, and 66% were in stage 3B/4 [2]. Approximately half of patients in stage 3B/4 receive chemotherapy to slow disease progression and prolong survival [2, 3]. Myelosuppressive chemotherapy regimens are frequently used to treat advanced NSCLC in US clinical practice [4] and typically include carboplatin, cisplatin, paclitaxel, pemetrexed, and gemcitabine [2]. Such agents are commonly associated with toxicities, typically managed with dose delays and reductions, in addition to prophylactic or supportive medications [5, 6]. Platinum-based chemotherapy is particularly associated with dose-limiting toxicities that require changes in dosing [7, 8]. Neutropenic complications (e.g., febrile neutropenia) often require delays or reductions in doses of myelosuppressive chemotherapy, which contribute to reduced relative dose intensity (RDI; typically defined as the ratio of the actual delivered chemotherapy dose to the planned dose) [9, 10].

In the adjuvant setting for NSCLC, reduced RDI and dose reductions have been associated with decreased survival [11]. However, the impact of dose changes should be considered in the context of overall health and comorbidities. There is limited information on the relationship between RDI and survival in patients with advanced NSCLC. A retrospective analysis of elderly patients with advanced NSCLC reported that patients who received RDI ≥ 80% experienced statistically higher response rates and overall survival (OS) than patients who received RDI < 80% [12]. To better understand chemotherapy intensity and its potential association with OS, we retrospectively analyzed dose delays, dose reductions, RDI, and the association between chemotherapy intensity and OS in patients with advanced/metastatic lung, breast, or ovarian cancer treated in a large US community-based oncology network setting. Prophylactic and reactive use of colony-stimulating factor (CSF) was also explored. Here, we report results from patients with advanced NSCLC. Results from patients with breast or ovarian cancer have been reported separately [13].

## Materials and methods

### Study design and data source

This was a retrospective cohort study of adult patients diagnosed with advanced lung cancer. Data were extracted from the McKesson Specialty Health/US Oncology iKnowMed™ database, a web-based electronic health record (EHR) system with oncology-specific data that includes records from more than 16 million patients and 900 US oncologist-users across nearly 230 practices. Medical chart review was not routinely conducted; however, it was performed as required to clarify data obtained from the EHR. The study protocol was submitted to the institutional review board and was exempted. The dosing analysis for this study was focused on the first-line chemotherapy course (i.e., index course) for each patient.

## Patients

### Inclusion criteria

Included patients were ≥ 18 years old, were diagnosed with advanced (stage 3 or 4) NSCLC, and had received first-line intravenous myelosuppressive platinum-based chemotherapy (Table 1) between January 2007 and December 2010 from oncology practices using the full EHR capacity of iKnowMed. Patients were also required to have had ≥ 4 visits in the US Oncology Network during the study period.

**Table 1:** Standard chemotherapy regimens

Chemotherapy regimen	Standard dose	Cycle length, days	Number of cycles
Carboplatin + paclitaxel	5 AUC/175 mg/m^2^	21	4
Pemetrexed + carboplatin	500 mg/m^2^/5 AUC	21	4
Bevacizumab + carboplatin + paclitaxel	15 mg/kg/5 AUC/175 mg/m^2^	21	4
Pemetrexed + cisplatin	500 mg/m^2^/75 mg/m^2^	21	4
Pemetrexed + bevacizumab + carboplatin	500 mg/m^2^/15 mg/kg/5 AUC	21	4
Carboplatin + gemcitabine	5 AUC/1000 mg/m^2^ × 2	21	4

*AUC* area under the plasma drug concentration-time curve

#### Exclusion criteria

Patients were excluded if they were enrolled in clinical trials for the treatment of advanced cancer at the time of enrollment, received only non-myelosuppressive chemotherapy agents during cycle 1, or received any oral myelosuppressive agents during the index course.

### Study outcomes

Patient data were examined for the 6-month period from initiation of chemotherapy for the estimation of dose delays, dose reductions, RDI, and incidence of RDI < 85%. The incidence of CSF use, prophylactic antimicrobial use, and grade 3/4 neutropenia was also examined. For estimation of OS, patients were followed up until death or the last visit date available.

As described for the part of this study that included patients with advanced breast or ovarian cancer [13], data were extracted from the iKnowMed database on the planned chemotherapy, the planned chemotherapy dose and schedule, the planned cycle length, the actual chemotherapy delivered, the actual chemotherapy dose and schedule, and the actual cycle length.

### Assessments

In this analysis, only myelosuppressive chemotherapy regimens with data for > 100 patients with NSCLC were included to ensure a sufficiently large sample size for analysis. Grade 3/4 neutropenia was defined as an absolute neutrophil count (ANC) of < 1000 cells/mm^3^ [14].

Colony-stimulating factor prophylaxis was defined as CSF use by day 5 of the chemotherapy cycle. Reactive treatment was defined as CSF use after day 5 of the chemotherapy cycle. Primary prophylaxis was defined as first CSF use within the first 5 days of chemotherapy cycle 1 of the index course; secondary prophylaxis was defined as first CSF use within the first 5 days in any subsequent cycle.

Chemotherapy dose delays, dose reductions, and RDI (Supplemental Table 1) were evaluated against standard chemotherapy regimens (Table 1). Information on standard regimens was extracted from the National Comprehensive Cancer Network guidelines version 1.2012, current at the time of this analysis [15], and from published clinical literature reporting efficacy and outcome results from phase 3 clinical studies.

Dose delays, dose reductions, and RDI were assessed over the first 6 months for each myelosuppressive agent that was administered as part of the standard chemotherapy regimen. Chemotherapy doses that were administered beyond 6 months were not considered in this study. For determination of dose delays and reductions, only intravenously administered myelosuppressive agents were considered. Dose delays were defined as a delay of ≥ 7 days from the standard regimen in any given chemotherapy cycle. A dose reduction was defined as a decrease of ≥ 15% in the chemotherapy dose relative to the standard (mg/m^2^) regimen for ≥ 1 myelosuppressive agent in any given cycle. Chemotherapy RDI was defined as the ratio of the delivered dose intensity to the standard dose intensity. The RDI definition for regimens containing multiple myelosuppressive agents was the mean of the individual RDI values for each myelosuppressive chemotherapy agent included in the regimen. If the chemotherapy dose or the number of cycles received within 6 months by a patient was higher than the standard dose or number of cycles, the RDI value determined for the chemotherapy course could exceed 100%. RDI < 85% is considered to be a clinically important variation from standard dosing as a part of a chemotherapy regimen [9, 16]; therefore, this value was used as the threshold to define reduced RDI. The termination date of the index course was defined as the date of the last chemotherapy administration plus the standard cycle length, the date of death, or the date of regimen change, whichever occurred first. Standard cycle length was determined for the corresponding standard chemotherapy regimen for the agent(s) in the first cycle. OS was defined as the time interval between the initiation of the first-line chemotherapy regimen and death or last follow-up.

Data were collected on eligible patients up to December 2010. Death events were defined by the Social Security Death Index and iKnowMed; patients without a death event were censored at the date of last observed visit.

### Statistical analyses

Statistical analyses were conducted as previously described for this study [13]. In brief, Kaplan-Meier unadjusted survival analyses and log-rank tests were performed to compare OS between patients with NSCLC who had chemotherapy dose delays ≥ 7 days versus < 7 days, dose reductions ≥ 15% versus < 15%, and RDI < 85% versus ≥ 85%.

Univariable Cox regression analyses were performed to determine the hazard ratio (HR) for each covariate. As known risk factors and based on clinical experience, the following variables were evaluated independently as survival predictors: dose delay ≥ 7 versus < 7 days, dose reduction ≥ 15% versus < 15%, RDI < 85% versus ≥ 85%, age at first visit, Eastern Cooperative Oncology Group performance status (ECOG PS) 1 or 2 versus 0, body surface area (BSA) > 2 versus ≤ 2 m^2^, baseline ANC < 1000 versus ≥ 1000, comorbidities ≥ 1 versus 0, hemoglobin < 12 versus ≥ 12 g/dL, and tumor subgroup (adenocarcinoma or other versus squamous). Multivariable Cox models included variables that were significant and marginally significant (*P* < 0.10) from the univariable analysis and were used to adjust for covariates. Univariable and multivariable models were developed as previously described for this study [13].

## Results

This study included 3866 patients with advanced NSCLC who met the inclusion criteria. Patient demographics and disease characteristics are listed in Table 2. The mean (SD) age was 66.8 years (10.0), 56.6% of patients were male, and 61.6% were ≥ 65 years old. A total of 892 patients (23.1%) had a BSA > 2 m^2^, and 1991 (51.5%) had an ECOG PS ≥ 1. Overall, 2088 patients (54.0%) had adenocarcinoma and 601 (15.6%) had squamous cell carcinoma; tumor subgroup was unknown or data were missing in 1050 patients (27.2%). The most common chemotherapy regimens were carboplatin + paclitaxel (1733 patients, 44.8%), pemetrexed + carboplatin (789 patients, 20.4%), and bevacizumab + carboplatin + paclitaxel (734 patients, 19.0%; Supplemental Table 2). At baseline, 86 patients (2.2%) had grade 3/4 neutropenia, defined as ANC < 1000 cells/mm^3^ (Table 2).

**Table 2:** Patient demographics and baseline characteristics

Characteristic^a^	Patients with NSCLC *N* = 3866
Mean (SD) age, years	66.8 (10.0)
Age group, years	
18–49	184 (4.8)
50–64	1299 (33.6)
65–74	1465 (37.9)
≥ 75	918 (23.8)
Men	2187 (56.6)
BSA > 2 m^2^	892 (23.1)
ECOG PS	
0	1484 (42.6)
1	1765 (50.7)
≥ 2	226 (6.5)
Unknown	8 (0.2)
Missing	383 (9.9)
Grade 3/4 neutropenia^b^	86 (2.2)
Tumor subgroup	
Adenocarcinoma	2088 (54.0)
Squamous cell carcinoma	601 (15.6)
Other	127 (3.3)
Unknown	426 (11.0)
Missing	624 (16.1)

*BSA* body surface area, *ECOGPS* Eastern Cooperative Oncology Group performance status, *NSCLC* non-small-cell lung cancer

^a^Data are expressed as *n* (%) unless otherwise noted

^b^Defined as absolute neutrophil count < 1000 cells/mm^3^

### Dose delays, dose reductions, and relative dose intensity

Dose delays, dose reductions, and reduced RDI were common. Overall, 32.4% of patients experienced a dose delay ≥ 7 days, 50.1% experienced a dose reduction ≥ 15%, and mean (SD) RDI across all regimens was 83.9% (28.5%), with 40.4% of patients receiving RDI < 85%. Dose delays, dose reductions, and reduced RDI were common even among young patients; among patients 18 to 49 years old, 27.7% (95% CI, 21.2%–34.3%) experienced a dose delay ≥ 7 days, 44.0% (36.8%–51.3%) experienced a dose reduction ≥ 15%, and 32.1% (25.3%–38.9%) experienced RDI < 85% (Supplemental Table 3). Reduced RDI was also common regardless of ECOG PS (ECOG PS ≥ 2, 56.2%; ECOG PS 0, 33.6%) and tumor subgroup (squamous cell carcinoma, 52.2%; adenocarcinoma, 36.0%; Supplemental Table 3). Mean (SD) RDI was 75.9% (34.4%) with carboplatin + paclitaxel, 95.0% (14.9%) with pemetrexed + carboplatin, and 87.1% (28.0%) with bevacizumab + carboplatin + paclitaxel (Supplemental Table 2).

### Neutropenia and supportive care

Among 3408 patients with evaluable ANC data, 24.4% experienced grade 3/4 neutropenia. Prophylaxis with CSFs or antimicrobials was uncommon; 18.3% received primary prophylaxis with CSFs, 6.3% received secondary prophylaxis with CSFs, and 31.0% received prophylaxis with oral antimicrobials (Table 3).

**Table 3:** CSF and antibiotic use

Characteristic, *n* (%)	Patients with NSCLC*N* = 3866
CSF use	
Primary prophylaxis^a^	709 (18.3)
Secondary prophylaxis^b^	242 (6.3)
Treatment^c^	636 (16.5)
Prophylactic oral antibiotics	1200 (31.0)

*CSF* colony-stimulating factor, *NSCLC* non-small-cell lung cancer

^a^First receipt of CSF within first 5 days of first cycle of the index course

^b^First receipt of CSF within first 5 days of subsequent cycles of the index course

^c^First receipt of CSF in any given cycle after cycle day 5

### Overall survival

**Fig. 1:**
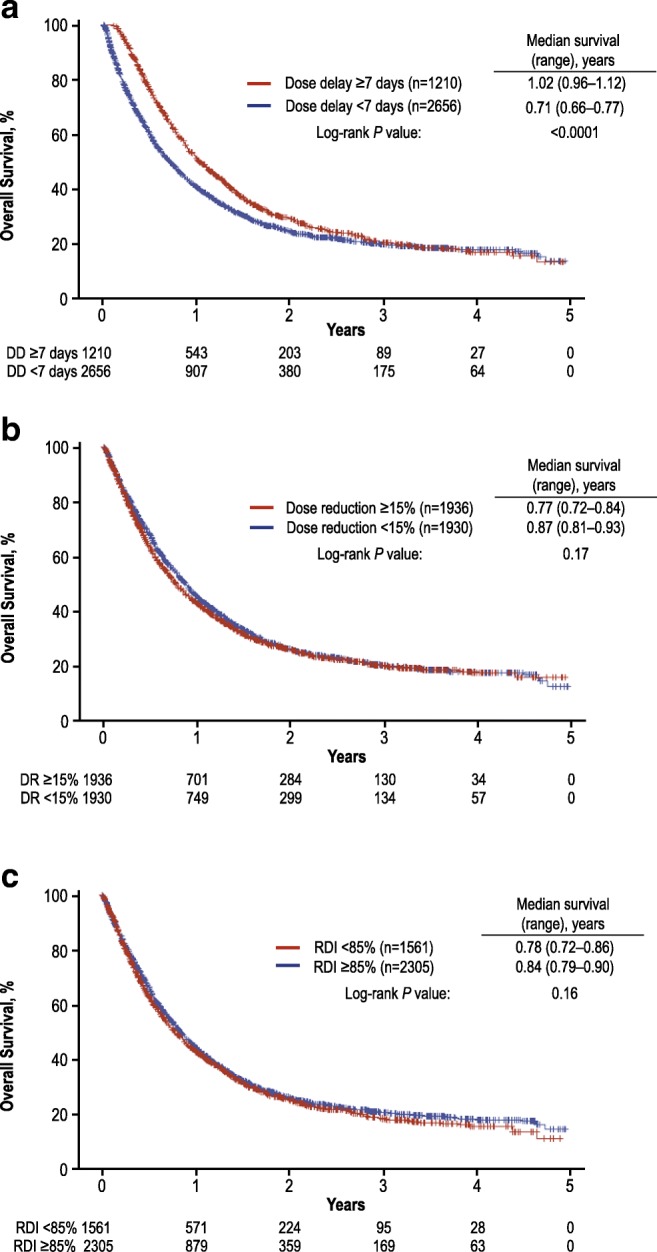
Overall survival for all chemotherapy regimens by **a** dose delay, **b** dose reduction, and **c** RDI among patients with NSCLC. *DD* dose delay, *DR* dose reduction, *NSCLC* non-small-cell lung cancer, *RDI* relative dose intensity

All 3866 patients with NSCLC were included in the OS analysis. At the time of the analysis, a total of 2674 patients (69%) had died. When OS was stratified by measures of chemotherapy intensity (dose delays < 7 versus ≥7 days, dose reductions < 15% versus ≥ 15%, and RDI < 85% versus ≥ 85%), significant differences in OS were observed only for dose delays (Fig. 1). Median (95% CI) OS was 1.02 years (0.96–1.12) for patients with dose delays ≥ 7 days and 0.71 years (0.66–0.77; log-rank *P* < 0.0001) for patients with dose delays < 7 days.

In the univariable analysis, dose delays ≥ 7 days (HR, 0.776 [95% CI, 0.715–0.841]) were significantly associated with a 22.4% reduction in the risk of death (Supplemental Table 4). Other measures of chemotherapy intensity (RDI and dose reductions) were not significantly associated with OS. ECOG PS 1 versus 0 (HR, 1.364 [95% CI, 1.255–1.483]) and 2 versus 0 (HR, 1.766 [1.498–2.081]) and hemoglobin < 12 versus ≥ 12 g/dL (HR, 1.205 [1.111–1.308]) were significantly associated with increased risk of death, whereas adenocarcinoma versus squamous cell carcinoma tumor subgroup (HR, 0.762 [0.685–0.847]) was significantly associated with a decreased risk of death.

The multivariable models included 2848 patients with complete data for dose reductions (≥ 7 versus < 7 days), ECOG PS (1 versus 0; 2 versus 0), and hemoglobin (< 12 versus ≥ 12 g/dL) and 2127 patients with complete data for those parameters as well as tumor subgroup (adenocarcinoma versus squamous; other versus squamous; Table 4). After controlling for covariates (ECOG PS, hemoglobin, and tumor subgroup), multivariable analysis suggested that dose delay ≥ 7 days and increased RDI were significant predictors of longer OS (Table 4). Dose delay ≥ 7 days was significantly associated with a 29.0% decrease in risk of death (*P* < 0.0001). RDI ≥85% was significantly associated with a 17.6% decrease in risk of death (*P* = 0.0062).

**Table 4:** Multivariable Cox regression analysis of OS in patients with NSCLC^a^

Variable	HR (95% CI)	*P* value
RDI, < 85% vs ≥ 85%	1.176 (1.047–1.320)	0.0062
Dose delay, ≥ 7 vs < 7 days	0.710 (0.630–0.800)	< 0.0001
ECOG PS		
1 vs 0	1.316 (1.192–1.453)	< 0.0001
2 vs 0	1.654 (1.350–2.027)	< 0.0001
Hemoglobin, < 12 vs ≥ 12 g/dL	1.098 (0.993–1.213)	0.0686
Tumor subgroup		
Adenocarcinoma vs squamous	0.783 (0.698–0.877)	< 0.0001
Other vs squamous	0.932 (0.725–1.199)	0.5855

*ECOG PS* Eastern Cooperative Oncology Group performance status, *HR* hazard ratio, *NSCLC* non-small-cell lung cancer, *OS* overall survival, *RDI* relative dose intensity

^a^In the overall study cohort of 3866 patients, 2674 (69%) deaths were observed. This analysis includes 2848 patients who had complete data for dose reductions, ECOG PS, and hemoglobin and 2127 patients who had complete data for ECOG PS, hemoglobin, and tumor subgroup

## Discussion

Chemotherapy dosing patterns and their subsequent impact on outcomes in the real-world treatment of advanced NSCLC are not well understood. There are few chemotherapy options for patients with non-squamous subtypes of lung cancer; therefore, it is critical to optimize chemotherapy delivery for those who are treated. A recent retrospective evaluation of dose intensity for the treatment of a variety of non-metastatic cancers in the community setting (that also used the iKnowMed EHR database) found that dose delay ≥ 7 days, dose reduction ≥ 15%, and RDI < 85% were frequent, including among patients treated with myelosuppressive chemotherapy regimens for NSCLC [6]. As in the earlier study [6], chemotherapy dose delays, reductions, and reduced RDI were common in patients with advanced NSCLC in this study.

Multivariable analysis suggested that dose delay ≥ 7 days was significantly associated with a 29% decreased risk of death. The effect of dose delay ≥ 7 days on OS was maintained out to 3 years (Fig. 1a). This suggests that the effect of dose delay on OS was not simply a reduction in toxicity, which would have had only a short-term impact on OS. One possibility is that those patients who had a dose delay of ≥ 7 days may have had a more profound degree of cytopenias and other complications that resulted in treatment delay compared with patients who had blood cell count recovery and could proceed with chemotherapy on time but required dose reduction. In both cases, the patients had experienced dose-limiting toxicity from the treatment, and this may be a better surrogate of chemotherapy dose delivery on an individual basis than RDI. Dose reduction alone was not a significant predictor of survival in either univariable or multivariable analysis in patients with NSCLC. RDI < 85% was not significantly associated with an increased risk of death in the univariable analysis; multivariable analysis suggested that RDI < 85% was significantly associated with an 18% increased risk of death. These seemingly conflicting results between dose delay ≥ 7 days and RDI < 85% may have been related to multiple factors, including differences in the definitions used: RDI is a composite measure of both dose delays and reductions that compares the full delivered dose of a complete chemotherapy with the standard dose within a 6-month period for a given chemotherapy standard regimen, whereas dose delay was calculated as a delay of ≥ 7 days in any given chemotherapy cycle from the standard regimen. Potential confounders, such as comorbidities, may also affect the results; however, they were not examined in this study. These results may also reflect physicians’ comfort with dose delays in a subset of patients to refine treatment management. For example, a patient who had a dose delayed to manage hematologic toxicity, but who was subsequently able to continue with the same dose and complete therapy, may have improved outcomes compared with a person whose dose was reduced or discontinued owing to comorbidities or other risk factors for death.

Dose delays, dose reductions, and reduced RDI were also common among younger patients with NSCLC (i.e., 18–49 years old); however, the reasons for this pattern in younger patients (e.g., pressure to continue to work, family duties, travel, adjustment to a new life-limiting diagnosis) may differ from the reasons in older patients (e.g., toxicity, comorbidity) [9], and reasons for dose delays in this study are unknown. The measures of chemotherapy intensity varied between the NSCLC subgroups, with dose delays, dose reductions, and reduced RDI being more frequent in patients with squamous cell carcinoma than adenocarcinoma. Patients with squamous cell carcinoma may have worse prognosis because of the increased likelihood of smoking, paraneoplastic syndrome, and ineligibility for many targeted therapies relative to patients with adenocarcinoma [17, 18].

Patterns for these measures of chemotherapy intensity also varied widely across chemotherapy regimens. Dose delays, dose reductions, and reduced RDI were more frequent in patients receiving treatment with carboplatin + gemcitabine or carboplatin + paclitaxel than the other chemotherapy regimens analyzed in this study. Pemetrexed + carboplatin had the highest mean RDI observed. Pemetrexed + bevacizumab + cisplatin had the lowest frequency of reduced RDI; pemetrexed + cisplatin had the lowest frequency of dose delays and dose reductions.

High rates of severe neutropenia (24.4%) were observed. Primary prophylaxis with CSF, which can help maintain the chemotherapy dose intensity [9], was received by 18.3% of patients, which may have affected mortality in this patient population.

Our results demonstrating the relationship between RDI < 85% and an increased risk of death in the multivariable analysis are consistent with results from an earlier study that found significantly reduced OS (7 versus 10 months) in patients with advanced NSCLC who received RDI < 80% versus RDI > 80% [12]. They are also similar to those from another study in patients with advanced epithelial ovarian cancer treated with platinum-based chemotherapy in which delivered RDI < 85% was associated with significantly reduced OS compared with delivered RDI > 85% [19]. In contrast, another study reported no difference in OS for patients with advanced NSCLC stratified by RDI < 90% versus ≥ 90%, which may be due to differences in the RDI thresholds (90% versus 85% in the present study), type of analysis (landmark analysis versus log-rank, respectively), and minimum number of cycles of chemotherapy received for inclusion in the analysis (1 versus 4) [20].

Compared with the present study, an earlier study conducted in patients receiving adjuvant or neoadjuvant chemotherapy for stage 1–3A NSCLC found higher rates of RDI < 85% (79.3% versus 53.4% in the present study), dose delay ≥ 7 days (63.9% versus 39.1%), and dose reductions ≥ 15% (83.6% versus 60.5%) for carboplatin plus paclitaxel [6]. These results suggest that physicians are more likely to decrease dose intensity in patients with less severe disease, hence confounding the results of this retrospective study.

Limitations of this study included the potential for selection bias due to the retrospective design and the possibility that inadequate or inaccurate codes could have introduced misclassification bias. Additionally, any services, medications, and procedures provided outside the McKesson Specialty Health/US Oncology Network were not captured by the iKnowMed EHR database and could not be ascertained in the study. We also did not have documentation regarding reasons for dose delays or reductions. This study relied on coded data fields in the iKnowMed database rather than on data extracted from medical charts, limiting our ability to determine certain clinical characteristics (e.g., histopathologic classification, comorbidities) and understand reasons for dose delays and reductions. There was no control for comorbid illness, which limited the ability to determine whether higher RDI was reflective of a healthier patient population and whether comorbid illness confounded CSF use and OS. The treatment patterns and clinical outcomes reflect the specific study time frame (2007–2010); extrapolation to current treatments is limited by changes in therapy since that time.

In summary, multivariable analysis in this study suggested that dose delay ≥ 7 days was significantly associated with a decreased risk of death, whereas RDI < 85% was significantly associated with an increased risk of death. The finding that dose reduction alone was not a significant predictor of survival warrants further investigation. These results suggest that different measurements of dose variation may have different sensitivity in predicting prognosis. Understanding the complex effect of dose intensity on outcomes will be important for managing toxicities and improving survival; research studies such as this one can inform decisions about supportive care, specifically CSF use, in patients with metastatic cancer. Further studies should investigate reasons for dose delays and reductions and also consider other potential confounders, including comorbid illness and socioeconomic factors.

## Electronic supplementary material

ESM 1 (PDF 90 kb).
