# Supplementary material for: Relative dose intensity of first-line chemotherapy and overall survival in patients with advanced non-small-cell lung cancer
Source: Support Care Cancer. 2019 Jun 7;28(2):925–32. doi: 10.1007/s00520-019-04875-1 (PMC6954126; doi:10.1007/s00520-019-04875-1)
Supplement: Supplementary file 1 — (PDF 90 kb). [file 520_2019_4875_MOESM1_ESM.pdf]

Supplemental Data

Supplemental Table 1. Definitions of Study Endpoints

**Study Endpoint**	**Definition**
RDI	Ratio of delivered dose intensity to standard dose intensity using standard regimen information
Delivered dose intensity	Ratio of total delivered dose over the chemotherapy course (mg/m^2) to actual time to complete chemotherapy or standard time to complete chemotherapy, whichever was longer (days)
Standard dose intensity	Ratio of standard total dose (mg/m^2) to standard time to complete chemotherapy (days)
Delivered total dose	Total amount of chemotherapy actually administered over chemotherapy course, based on actual administered number of chemotherapy cycles and actual administered dose in each of those cycles
Actual time to chemotherapy	Observed number of days between index date and termination date of index course
Standard time to complete chemotherapy	(Standard cycle length, days) × (standard number of cycles)
Standard total dose	(Standard dose, mg/m^2) × (standard number of cycles)
Dose delay	Delay ≥7 days from the standard regimen in any given cycle
Dose reduction	Decrease in chemotherapy dose ≥15% relative to standard (mg/m^2) for ≥1 myelosuppressive agent in any given cycle

RDI=relative dose intensity.

Supplemental Table 2. RDI, Dose Delays, and Dose Reductions by Standard Regimen

NSCLC Chemotherapy Regimen, %	Mean (SD) RDI	RDI <85% (95% CI)	Dose Delay ≥7 Days (95% CI)	Dose Reduction ≥15% (95% CI)
Carboplatin + paclitaxel (n=1733)	75.9 (34.4)	53.4 (51.0–55.7)	39.1 (36.9–41.4)	60.5 (58.2–62.8)
Pemetrexed + carboplatin (n=789)	95.0 (14.9)	23.2 (20.4–26.3)	22.2 (19.4–25.2)	37.5 (34.2–41.0)
Bevacizumab + carboplatin + paclitaxel (n=734)	87.1 (28.0)	36.8 (33.4–40.3)	34.3 (31.0–37.8)	46.9 (43.3–50.5)
Pemetrexed + cisplatin (n=317)	91.0 (12.2)	22.7 (18.1–27.3)	18.9 (15.0–23.6)	25.9 (21.1–30.7)
Pemetrexed + bevacizumab + carboplatin (n=163)	92.7 (19.1)	22.7 (16.9–29.7)	26.4 (20.2–33.6)	42.9 (35.6–50.6)
Carboplatin + gemcitabine (n=130)	86.8 (29.8)	56.9 (48.3–65.1)	35.4 (27.7–43.9)	73.1 (64.9–80.0)

NSCLC=non–small-cell lung cancer; RDI=relative dose intensity.

Supplemental Table 3. RDI, Dose Delays, and Dose Reductions Among Subgroups of Patients With NSCLC

Patient Characteristics, % (95% CI)	Mean RDI	RDI <85%	Dose Delay ≥7 Days	Dose Reduction ≥15%
Age group, years				
18–49 (n=184)	86.8 (82.9–90.7)	32.1 (25.3–38.9)	27.7 (21.2–34.3)	44.0 (36.8–51.3)
50–64 (n=1299)	88.8 (87.3–90.3)	32.2 (29.6–34.7)	27.6 (25.1–30.0)	41.5 (38.8–44.2)
65–74 (n=1465)	85.0 (83.6–86.5)	38.6 (36.1–41.1)	33.0 (30.6–35.5)	48.9 (46.4–51.5)
≥75 (n=918)	74.8 (72.9–76.7)	56.5 (53.3–59.8)	39.3 (36.2–42.5)	65.3 (62.2–68.3)
BSA, m^2				
≤2 (n=2973)	83.6 (82.6–84.6)	40.5 (38.8–42.3)	32.8 (31.1–34.5)	50.0 (48.2–51.8)
>2 (n=892)	85.1 (83.3–86.9)	39.9 (36.7–43.1)	31.4 (28.3–34.4)	50.4 (47.1–53.7)
ECOG PS				
0 (n=1484)	87.8 (86.4–89.1)	33.6 (31.2–36.0)	31.4 (29.0–33.8)	44.7 (42.2–47.3)
1 (n=1765)	81.6 (80.2–83.0)	44.3 (42.0–46.6)	34.1 (31.8–36.3)	53.1 (50.8–55.4
≥2 (n=226)	75.1 (71.4–78.8)	56.2 (49.7–62.7)	30.1 (24.1–36.1)	62.8 (56.5–69.2)
Unknown (n=8)	94.4 (69.9–118.8)	12.5 (0–42.1)	50.0 (5.3–94.7)	12.5 (0–42.1)
Missing (n=383)	84.9 (82.2–87.6)	39.7 (34.8–44.6)	30.0 (25.4–34.6)	50.1 (45.1–55.2)
Tumor subgroup				
Adenocarcinoma (n=2085)	86.2 (85.1–87.3)	36.0 (33.9–38.0)	30.7 (28.7–32.7)	46.9 (44.7–49.0)
Squamous cell carcinoma (n=600)	78.4 (75.8–81.1)	52.2 (48.2–56.2)	40.3 (36.4–44.3)	59.0 (55.1–63.0)
Other (n=127)	83.4 (78.4–88.4)	38.6 (30.0–47.2)	36.2 (27.8–44.7)	56.7 (48.0–65.4)
Unknown (n=425)	78.4 (75.3–81.5)	48.9 (44.2–53.7)	35.8 (31.2–40.3)	54.8 (50.1–59.6)
Missing (n=629)	85.6 (83.5–87.8)	38.3 (34.5–42.1)	27.7 (24.2–31.2)	47.7 (43.8–51.6)

BSA=body surface area; ECOG PS=Eastern Cooperative Oncology Group performance status; NSCLC=non–small-cell lung cancer; RDI=relative dose intensity.

Supplemental Table 4. Univariable and Multivariable Cox Regression Analysis for Overall Survival in NSCLC

	Univariable			Multivariable	
**Variable**	**n**	**HR (95% CI)**	***P* Value**	**HR (95% CI)**	***P* Value**
RDI, <85% vs ≥85%	3866	1.057 (0.979–1.142)	0.16	1.176 (1.047–1.320)	0.0062
Dose delay, ≥7 vs <7 days	3866	0.776 (0.715–0.841)	<0.0001	0.710 (0.630–0.800)	<0.0001
Dose reduction, ≥15% vs <15%	3866	1.055 (0.978–1.138)	0.17		
Age at first visit, years (continuous)	3549	1.002 (0.998–1.006)	0.27		
ECOG PS	3475				
1 vs 0		1.364 (1.255–1.483)	<0.0001	1.316 (1.192–1.453)	<0.0001
2 vs 0		1.766 (1.498–2.081)	<0.0001	1.654 (1.350–2.027)	<0.0001
BSA >2 vs ≤2 m^2	3866	0.988 (0.903–1.080)	0.78		
Baseline ANC, <1000 vs ≥1000	3593	0.919 (0.742–1.138)	0.44		
Comorbidity, ≥1 vs 0	3866	1.023 (0.942–1.110)	0.59		
Hemoglobin, <12 vs ≥12 g/dL	3663	1.205 (1.111–1.308)	<0.0001	1.098 (0.993–1.213)	0.0686
Tumor subgroup	2812				
Adenocarcinoma vs squamous		0.762 (0.685–0.847)	<0.0001	0.783 (0.698–0.877)	<0.0001
Other vs squamous		0.929 (0.740–1.166)	0.53	0.932 (0.725–1.199)	0.5855

ANC=absolute neutrophil count; BSA=body surface area; ECOG PS=Eastern Cooperative Oncology Group performance status; HR=hazard ratio; NSCLC=non–small-cell lung cancer; RDI=relative dose intensity.
